# Executive Dysfunction After COVID-19 in an Older Adult With Type 1 Diabetes: A Case of Insulin Pump Discontinuation

**DOI:** 10.7759/cureus.93554

**Published:** 2025-09-30

**Authors:** Yuya Asano, Takahiro Kamihara, Takuya Omura

**Affiliations:** 1 Department of Medical Education, National Center for Geriatrics and Gerontology, Obu, JPN; 2 Department of Cardiology, National Center for Geriatrics and Gerontology, Obu, JPN; 3 Department of Metabolic Research, National Center for Geriatrics and Gerontology, Obu, JPN; 4 Department of Diabetes and Endocrinology, National Center for Geriatrics and Gerontology, Obu, JPN

**Keywords:** covid-19, diabetic ketoacidosis, dysgeusia, executive dysfunction, frailty, insulin pump, older adult diabetes, type 1 diabetes mellitus, zinc

## Abstract

Continuous subcutaneous insulin infusion (CSII) and multiple daily injections (MDIs) are the two main approaches to intensive insulin therapy. CSII provides flexible basal-bolus adjustments and can improve glycemic variability, but it requires preserved executive function for tasks such as infusion set replacements, bolus programming, and troubleshooting in response to pump alarms. In contrast, MDI involves more injections but is simpler to operate and less cognitively demanding. Advanced diabetes technologies such as CSII require more than intact cognition; they rely on real-world executive function and consistent self-management, especially in older adults with type 1 diabetes mellitus (T1DM). Even common infections, such as COVID-19, can unmask functional vulnerabilities that compromise the safety of such therapies. Post-infectious changes in attention, decision-making, or nutrition may precede detectable impairments on tools like the Montreal Cognitive Assessment (MoCA). Here, we describe the case of a 73-year-old woman with a 28-year history of T1DM who had been independently and stably using CSII. Following a COVID-19 infection, she developed fatigue, dysgeusia, and appetite loss. Despite a high MoCA-J score (29/30), she experienced recurrent episodes of diabetic ketoacidosis (DKA). Direct pump log review was not possible. Missed mealtime boluses were inferred from preadmission self-monitoring of blood glucose patterns showing frequent missing entries and, when available, marked postprandial hyperglycemia, together with the patient’s inconsistent recollection of bolus administration. Although these indirect findings could not definitively prove pump errors, they suggested impaired executive function. Laboratory testing showed zinc deficiency (63 µg/dL), and supplementation improved taste and oral intake. Due to persistent pump-related errors and reduced confidence, her insulin regimen was transitioned to MDIs, resulting in stable glycemic control without further DKA, although she required one additional hospitalization for post-COVID-19 anorexia and nutritional decline over a three-month follow-up period. In older adults with T1DM, even seemingly mild illnesses such as COVID-19 can trigger disproportionate functional decline. Clinicians should recognize that executive dysfunction may precede cognitive test abnormalities. Incorporating functional assessments and simplifying therapy when indicated may help prevent severe outcomes such as DKA in this vulnerable population.

## Introduction

Older adults with type 1 diabetes mellitus (T1DM) face unique challenges related to age-associated cognitive decline, polypharmacy, and diminished physiological reserve. As continuous subcutaneous insulin infusion (CSII) becomes more common in this population, its safe and effective use relies heavily on preserved executive function, working memory, and attentional capacity [1,2]. Even subtle deficits in these domains can compromise device safety and adherence, increasing the risk of complications such as diabetic ketoacidosis (DKA).

The COVID-19 pandemic has introduced additional complexity to diabetes management in older adults. While acute metabolic consequences, such as hyperglycemia and DKA, are well recognized, post-COVID-19 sequelae, including executive dysfunction (“brain fog”), dysgeusia, and micronutrient imbalances (notably hypozincemia), are increasingly reported [3]. These conditions can negatively impact nutritional intake and the ability to manage insulin pump therapy, even in individuals with previously intact self-care skills.

Importantly, such functional deficits may precede detectable changes on standard cognitive screening tools such as the Montreal Cognitive Assessment (MoCA), creating a diagnostic gap [4]. Despite apparently normal test scores, patients may exhibit executive dysfunction in real-world tasks such as insulin dosing, alarm response, or coordinating meals.

We present the case of an older adult woman with longstanding T1DM who developed post-COVID-19 dysgeusia, zinc deficiency, and subtle executive dysfunction, ultimately resulting in insulin pump mismanagement and recurrent DKA. This case highlights the importance of supplementing cognitive screening with real-world functional and nutritional assessment when evaluating the continued appropriateness of CSII therapy in older adults recovering from COVID-19.

## Case presentation

A 73-year-old woman with a 28-year history of T1DM, diagnosed at age 45, was admitted with recurrent DKA. Her medical history included osteoporosis, hypothyroidism, and hyperlipidemia. She had no prior episodes of DKA and had independently managed her diabetes with CSII for one year (after previously being on multiple daily injections, MDIs), initiated to improve glycemic variability and reduce hypoglycemia, without previous complications. Before COVID-19 infection, her HbA1c was generally in the 8% range, and continuous glucose monitoring (CGM)-derived time in range was approximately 50-60%. She lived alone without caregiver support, precluding collateral cognitive reporting. Following SARS-CoV-2 infection in December 2023, she required two hospitalizations for DKA (December 2023 and January 2024), followed by a third hospitalization in March 2024 for post-COVID-19 anorexia and nutritional decline without DKA.

On December 21, 2023, after attending a social gathering, she developed a dry cough, anorexia, and polydipsia. She tested positive for SARS-CoV-2 on December 27 and was admitted to the hospital the same day with persistent fatigue, decreased appetite, dehydration, and fluctuating glycemic control, leading to DKA.

On her first admission, she was alert and fully oriented. Physical examination findings included height, 146.5 cm; weight, 46.6 kg (body mass index: 21.7 kg/m²); temperature, 37.2°C; blood pressure, 110/58 mmHg; heart rate, 105 beats/minute; and oxygen saturation, 96% on room air. Laboratory findings are summarized in Table 1.

**Table 1:** Laboratory data at the first admission. All parameters were obtained at the first admission, except serum zinc, which was measured at the second admission.

Parameter	Patient value	Unit	Reference range
Hematology			
White blood cell counts	14,300	/μL	3,300–8,600
Neutrophils	83.9	%	38–68
Lymphocytes	9.5	%	27–47
Monocytes	6.3	%	2.0–8.0
Eosinophils	0.1	%	0.0–7.0
Basophils	0.2	%	0.0–1.0
Red blood cell counts	4.21 × 10⁶	/μL	4.35–5.55 × 10⁶
Hemoglobin	13.1	g/dL	11.6–14.8
Hematocrit	40.8	%	35.1–44.4
Platelet	27.6 × 10⁴	/μL	15.8–34.8 × 10⁴
Biochemistry			
Total protein	7.8	g/dL	6.6–8.1
Albumin	4.6	g/dL	4.1–5.1
Aspartate aminotransferase	29	IU/L	13–30
Alanine transaminase	31	IU/L	7.0–23
Amylase	35	IU/L	44–132
Creatine kinase	145	IU/L	41–153
Glucose	340	mg/dL	73–109
Urea nitrogen	30	mg/dL	8.0–20
Creatinine	0.7	mg/dL	0.46–0.79
Total ketone bodies	8978	μmol/L	0.0–129
Sodium	139	mEq/L	138–145
Potassium	4.5	mEq/L	3.6–4.8
Zinc	63	μg/dL	80–130
HbA1c	9.4	%	4.9–6.0
Serology			
C-reactive protein	1.35	mg/dL	0.00–0.14
Venous blood gas			
pH	7.22		7.32–7.42
Bicarbonate	15	mmol/L	21–29
Nasal swab			
SARS-CoV-2 Ag (cutoff index)	5,324	Index	0.0–0.9

Upper gastrointestinal endoscopy demonstrated superficial erosive gastritis, and esomeprazole was initiated. She was treated according to the local DKA protocol, including intravenous insulin, isotonic fluids, and potassium supplementation as clinically indicated, followed by a five-day course of oral molnupiravir.

Although she achieved clinical stabilization with acute management during both the first and second hospitalizations, subsequent self-management revealed problems. A retrospective analysis of her sensor glucose data suggested multiple missed mealtime boluses. Additionally, the patient exhibited poor recollection regarding pump operation, and it was later discovered that the insulin pump device had been lost during her second emergency transport, precluding direct review of pump logs. Instead, pump mismanagement was inferred from self-monitoring of blood glucose (SMBG) records showing frequent omissions and episodes of marked postprandial hyperglycemia when data were available. Moreover, although the patient had previously been able to clearly report her bolus doses, after COVID-19, she became unable to provide consistent or accurate information regarding whether boluses had been administered or the units delivered.

In addition, CGM data obtained during the subsequent observation period (February 8-20, 2024) demonstrated multiple rapid postprandial excursions. Although not definitive evidence of pump errors, these findings were interpreted as strongly suggestive of executive dysfunction in real-world self-management.

Her Montreal Cognitive Assessment-Japanese version (MoCA-J) scores were 23/30 in May 2023 (at which time she was still managing CSII without apparent difficulties), 29/30 in January 2024, and 25/30 in April 2024. Notably, although her MoCA-J score was lower in May 2023, no clear self-management problems were evident at that time, whereas episodes of pump mismanagement emerged when her score was highest in January 2024, indicating a dissociation between cognitive screening scores and functional self-management capacity.

The patient also reported persistent dysgeusia, which likely contributed to reduced caloric intake. Zinc supplementation (initially with polaprezinc, later switched to zinc acetate hydrate) resulted in gradual improvement in taste perception and appetite.

**Figure 1:**
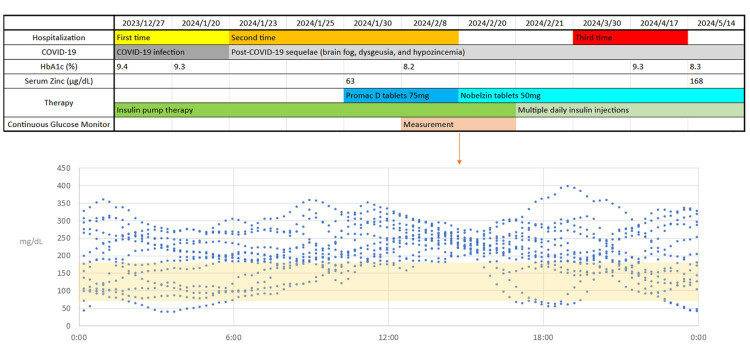
Clinical course and glycemic control. Overlaid continuous glucose monitoring traces from February 8 to February 20, 2024, showing daily glucose profiles during continuous monitoring.

She was discharged on CSII after both the first and second admissions. However, due to persistent CSII-related errors and rising frustration with the device, the care team transitioned her to MDI (Figure 1). CGM data were continuously collected between February 8 and 20, 2024, during CSII therapy before transition to MDI. Figure 1 shows the superimposed daily glucose profiles from this period.

Treatment: pharmacological and non-pharmacological interventions

At admission for DKA, the patient received standard treatment, including continuous intravenous insulin infusion and isotonic saline, with close monitoring of electrolyte levels and acid-base balance. In light of her SARS-CoV-2 infection and high-risk profile, including T1DM, advanced age, and recent signs of functional decline, oral molnupiravir was administered at 800 mg twice daily for five days. Her COVID-19 course remained clinically mild, without respiratory deterioration.

Before admission, her body weight was approximately 48 kg, which had declined to 46.6 kg on admission, consistent with her reported anorexia. To evaluate her anorexia and weight loss, an upper endoscopy was performed, revealing erosive gastritis. Treatment with esomeprazole (20 mg daily) led to improvement in dyspeptic symptoms.

Her serum zinc level was markedly reduced at 63 µg/dL (reference range = 80-130 µg/dL), consistent with hypozincemia. Dysgeusia was attributed in part to this deficiency. Zinc was initially supplemented with oral polaprezinc (75 mg twice daily) and later switched to zinc acetate hydrate (50 mg elemental zinc/day), which was associated with gradual improvement in taste and appetite; however, she was rehospitalized on March 30, 2024, for anorexia without DKA.

Despite improved metabolic control, she continued to struggle with CSII use, including missed boluses and failure to respond to pump alarms, which were interpreted as manifestations of executive dysfunction not detected on cognitive screening tests. After her second discharge, and with the patient’s agreement, her insulin therapy was changed to MDI. This change led to improved treatment adherence and no further DKA episodes.

Throughout her hospitalization, the patient received multidisciplinary care involving endocrinology, geriatrics, gastroenterology, and nutrition teams. Her care included structured diabetes education, psychosocial support, and individualized therapy modifications.

Outcome and follow-up

After her second discharge, the patient was followed closely in outpatient settings through a multidisciplinary approach. After that, she experienced no recurrence of DKA. The transition to MDI therapy resulted in improved adherence, enhanced confidence, and reduced treatment-related anxiety, likely due to the simplified regimen and the patient’s prior familiarity with MDI before initiating CSII.

Outpatient follow-up included CGM, nutritional counseling, and serial neurocognitive assessments. Zinc supplementation was continued, leading to an increase in serum zinc level from 63 μg/dL to 168 μg/dL, above the reference range (80-130 μg/dL). This elevation was associated with improved taste and appetite.

Interestingly, although her MoCA-J score declined from 29 in January 2024 to 25 in April 2024, her CSII-related self-care failures had occurred when her cognitive score was highest, underscoring a disconnect between structured cognitive testing and real-world functional performance.

Gastrointestinal symptoms were well controlled with continued esomeprazole, and a repeat upper endoscopy was scheduled for six months post-discharge to monitor mucosal healing. The patient made a full recovery from COVID-19, with no lingering respiratory or neuropsychiatric symptoms. By the end of the observation period, she had regained her prior level of independence, achieved stable glycemic control, and expressed satisfaction with her MDI regimen under the guidance of her endocrinologist and care team.

## Discussion

This case highlights the nuanced interplay between post-COVID-19 functional sequelae, cognitive integrity, and advanced diabetes management in an older adult with T1DM. Despite prior stability on CSII, the patient developed executive dysfunction and dysgeusia after SARS-CoV-2 infection, ultimately impairing her ability to safely operate insulin pump therapy. These functional impairments occurred even when her MoCA-J score was high (29/30), underscoring the potential disconnect between conventional cognitive screening tools and actual self-management capacity.

Cognitive function and device use

The safe and effective use of CSII requires intact executive functions such as prospective memory, attention regulation, and rapid decision-making in response to device alerts. While MoCA is a widely used screening tool in older adults, it may lack the sensitivity to detect subtle executive deficits that compromise daily self-care. In this patient, missed boluses and unacknowledged alarms occurred during a period of near-perfect cognitive screening scores, illustrating how performance-based dysfunction may precede detectable changes in structured assessments.

The CGM traces obtained before MDI transition (February 8-20, 2024, while on CSII) revealed repeated postprandial hyperglycemia, supporting our interpretation that executive dysfunction impaired insulin dosing. After switching to MDI on February 21, glycemic control stabilized, and no further DKA occurred.

Emerging evidence on post-COVID-19 cognitive changes, including the so-called “brain fog,” describes impairments in attention, processing speed, and mental clarity. These manifestations often resemble executive dysfunction and have been reported following infections, chemotherapy, and critical illness. In older adults with T1DM, even modest cognitive decline can have disproportionately serious consequences given the complexity of insulin dosing and device management [5].

Dysgeusia, hypozincemia, and nutritional risk

The patient’s dysgeusia contributed to appetite loss and erratic carbohydrate intake, which likely exacerbated glycemic instability. Her confirmed hypozincemia may have been both a cause and a consequence of altered taste perception and impaired nutritional recovery.

To our knowledge, this is among the first reports to describe persistent dysgeusia and hypozincemia as contributing factors in the failure of CSII management in an older adult with T1DM recovering from COVID-19. While cognitive and technical competencies are often emphasized in assessments of diabetes technology readiness, this case illustrates how nutritional impairments and sensory changes, specifically taste disturbance (dysgeusia), particularly in older individuals with limited physiological reserve, can critically compromise real-world self-management.

Clinical implications

This case highlights the limitations of relying solely on cognitive screening tools such as the Mini-Mental State Examination or MoCA when assessing technology readiness in older adults with T1DM. Although this patient’s MoCA score remained high, her missed boluses and lack of response to insulin pump alarms revealed executive difficulties not captured by formal testing.

The later decline to 25/30 in April 2024 may have been influenced not only by underlying cognitive changes but also by the effects of hospitalization and repeated DKA, both of which are known to transiently impair cognitive function. This temporal pattern emphasizes that structured cognitive screening tools may not fully capture real-world executive dysfunction in older adults with T1DM.

A more accurate evaluation of self-care capacity may require a combination of real-world data (e.g., insulin delivery records, alarm responsiveness), caregiver observations, and interdisciplinary input. Such approaches are particularly relevant in older individuals, where even subtle cognitive or sensory impairments may translate into disproportionate clinical risks.

Context within the literature

This case expands on emerging literature highlighting the impact of post-COVID-19 functional impairments in vulnerable groups, particularly those with older adult diabetes [6]. It illustrates how seemingly subtle deficits, such as impaired taste, nutritional instability, or mild executive dysfunction, can have disproportionate consequences when combined with age-related physiological decline, infection-related metabolic stress, and the demands of complex treatment regimens such as CSII.

While existing guidelines for diabetes technology tend to emphasize cognitive testing and technical competency, this case illustrates the need to incorporate broader domains of risk. Routine evaluation of post-infectious functional status, micronutrient levels (especially zinc), and sensory changes (especially taste disturbance) may help detect early signs of decompensation. Importantly, it also reinforces that technology failure can occur even in patients with preserved cognitive screening scores, suggesting that performance-based and context-sensitive assessments are essential for guiding safe therapy choices in older adults.

These findings are consistent with prior reports of post-acute COVID-19 syndrome describing nutritional disturbances such as hypozincemia [3] and persistent cognitive dysfunction resembling executive impairment (“brain fog”) [5]. In addition, limitations of cognitive screening tools in detecting subtle executive dysfunction in older adults with type 1 diabetes have been highlighted [4], further supporting our interpretation.

A key limitation of this case is that direct pump log data were unavailable. Our interpretation, therefore, relied on SMBG patterns, subsequent CGM traces, and the patient’s inconsistent recollection of insulin dosing. While these indirect observations cannot definitively establish pump errors, they strongly suggest impaired executive function affecting diabetes self-management. Another limitation is that formal executive function tests were not conducted. Future evaluations should therefore incorporate objective executive function tests, such as the Trail Making Test, Stroop Test, or Clock Drawing Test, together with caregiver observations and functional assessments.

## Conclusions

This case highlights the critical role of functional assessment in evaluating the continued use of advanced diabetes technology among older adults recovering from COVID-19. Although standard cognitive screening results, such as a high MoCA-J score, appeared within normal limits, the patient’s mismanagement of insulin pump therapy and repeated episodes of DKA revealed clinically significant impairments in executive and sensory function. Targeted interventions, including simplification of the insulin regimen from CSII to MDI and zinc repletion, proved effective in stabilizing glycemic control and restoring patient confidence, although one further hospitalization occurred due to post-COVID-19 anorexia without DKA. Notably, these improvements were achieved by addressing vulnerabilities not detected through formal cognitive testing, such as dysgeusia, nutritional instability, and executive dysfunction. This case underscores the need for proactive, multidimensional evaluation of older adults with type 1 diabetes following infectious illness. Incorporating real-world performance indicators, caregiver input, and nutritional assessments may help identify functional decline that precedes measurable cognitive changes. Early recognition and individualized treatment adjustment can reduce the risk of acute complications and promote safer long-term self-management in this vulnerable population. This case suggests that executive dysfunction may precede detectable abnormalities on cognitive screening tools; however, it remains unclear whether executive dysfunction represents an independent and earlier factor compared with cognitive impairment. Further case accumulation and longitudinal studies will be necessary to clarify this relationship.

## References

[1] Grammes J, Küstner E, Dapp A (2020). Comparative characteristics of older people with type 1 diabetes treated with continuous subcutaneous insulin infusion or insulin injection therapy: data from the German/Austrian DPV registry. Diabet Med.

[2] Omura T, Tamura Y, Kodera R (2019). Oldest-old type 1 diabetes patient receiving insulin pump treatment with positive myeloperoxidase-antineutrophil cytoplasmic antibody complication: a case report. Geriatr Gerontol Int.

[3] Nalbandian A, Sehgal K, Gupta A (2021). Post-acute COVID-19 syndrome. Nat Med.

[4] Choe J, Kudrna R, Fonseca LM, Chaytor NS (2023). Usefulness of the Montreal Cognitive Assessment in older adults with type 1 diabetes. Diabetes Spectr.

[5] Graham EL, Clark JR, Orban ZS (2021). Persistent neurologic symptoms and cognitive dysfunction in non-hospitalized Covid-19 "long haulers". Ann Clin Transl Neurol.

[6] Omura T (2025). “Older adult diabetes”: a conceptual proposal for a distinct clinical entity. Diabetes Spectr.

